# Reduced TLR3 and TLR9 Expression in Epidermodysplasia Verruciformis: Evidence From a Comparative Skin Study

**DOI:** 10.1002/jmv.70696

**Published:** 2025-11-10

**Authors:** Luis Alberto Ribeiro Fróes, Cibele Conceição dos Apóstolos Pereira, Lana Luiza da Cruz Silva, Naiura Vieira Pereira, Walmar Roncalli Pereira de Oliveira, Mirian Nacagami Sotto

**Affiliations:** ^1^ Department of Pathology University of São Paulo Medical School São Paulo Brazil; ^2^ Department of Dermatology University of São Paulo Medical School São Paulo Brazil; ^3^ Tropical Medicine Institute University of São Paulo São Paulo Brazil

**Keywords:** Epidermodysplasia verruciformis, flat warts, innate immunity, toll‐like receptors, β‐human papillomavirus

## Abstract

Epidermodysplasia verruciformis (EV) is a rare genodermatosis characterized by lifelong β‐human papillomavirus (β‐HPV) persistence, extensive flat warts, and increased risk of cutaneous squamous cell carcinoma. While *TMC6, TMC8, and CIB1* mutations are recognized as genetic drivers, innate immune mechanisms contributing to HPV persistence remain incompletely defined. This study quantified the expression of Toll‐like receptors (TLRs) 3, 4, 5, and 9 in normal skin and flat warts from patients with EV and immunocompetent individuals without EV (NEV). We performed immunohistochemical analysis on 135 formalin‐fixed, paraffin‐embedded specimens using standardized digital morphometry of epidermal keratinocytes. EV patients exhibited significantly reduced TLR3 and TLR9 expression in both normal skin and flat warts relative to controls, whereas TLR4 and TLR5 levels were comparable. Notably, flat warts from NEV individuals showed marked TLR3 upregulation relative to matched normal skin, whereas this response was absent in EV patients. These findings are consistent with an EV‐associated epithelial innate‐sensing phenotype. Our data suggest innate immune deficiencies may interact with previously described keratinocyte abnormalities, amplifying local immune dysfunction. These findings provide a framework for investigating TLR‐based therapeutic approaches in EV.

## 1 Introduction

Toll‐like receptors (TLRs) are pattern‐recognition receptors that detect conserved microbial motifs and signal through MyD88‐ or TRIF‐dependent pathways; in particular, TLR3 recognizes mainly double‐stranded RNA, TLR4 recognizes lipopolysaccharide, TLR5 recognizes flagellin, and TLR9 recognizes unmethylated CpG DNA [1, 2, 3]. Human keratinocytes—key epithelial sentinels in skin—express TLR3, TLR4, TLR5, and TLR9 and mount ligand‐induced inflammatory programs in vitro, underscoring that TLR expression in the epidermis is biologically meaningful even outside professional immune cells [4].

Epidermodysplasia verruciformis (EV) is a rare genodermatosis with lifelong susceptibility to cutaneous β‐HPV infection and elevated squamous‐cell carcinoma risk on sun‐exposed skin [5]. EV results from biallelic loss of *TMC6/TMC8* or *CIB1*, with CIB1–EVER1–EVER2 forming a keratinocyte‐intrinsic complex for β‐HPV restriction [6, 7]. Genetic studies distinguish “typical” skin‐restricted EV from “atypical” EV with broader immunodeficiency [8]. RNA‐seq shows β‐HPV transcripts enriched in lesional versus matched normal skin, reinforcing EV as a cutaneous β‐HPV model [9].

Given the viral etiology of EV lesions and the role of TLRs in antiviral immunity, understanding TLR expression in these lesions may provide insights into disease pathogenesis. Studies outside EV show altered TLR expression in cutaneous viral lesions: common warts and molluscum contagiosum display increased epidermal TLR3/TLR9 versus normal skin [10]; verruca vulgaris shows elevated dermal TLR9/IRAK1 (interleukin‐1 receptor‐associated kinase 1) with increased plasmacytoid dendritic cells [11]. Moreover, EV shows altered epidermal differentiation compared with flat warts in non‐EV individuals, highlighting disease‐specific cutaneous programs [12].

While most TLR‐HPV literature focuses on mucosal sites [13, 14, 15], TLR expression patterns in EV lesions remain uncharacterized, limiting understanding of innate immune dysfunction and therapeutic targets. We hypothesized that EV lesions display distinct TLR expression patterns reflecting the unique environment permitting persistent β‐HPV infection. We therefore compared epidermal TLR3, TLR4, TLR5, and TLR9 expression in normal skin and flat warts from individuals with and without EV.

## 2 Materials and Methods

### 2.1 Study Design and Sample Selection

This retrospective comparative study was conducted at the Dermatopathology Laboratory, Hospital das Clínicas, Universidade de São Paulo. Formalin‐fixed, paraffin‐embedded (FFPE) skin samples were retrieved from institutional archives (2010–2024) and reviewed by two dermatopathologists. The study was approved by the Institutional Ethics Committee of Hospital das Clínicas, University of São Paulo, which waived informed consent due to exclusive use of archived, anonymized samples (approval number #50871415.2.0000.0068), in accordance with the Declaration of Helsinki.

Patients were classified as having epidermodysplasia verruciformis (EV) using strict clinicopathological criteria, including the characteristic clinical phenotype, histopathological features showing EV‐type cytopathic changes [16]. compatible with β‐HPV infection, and longitudinal follow‐up confirming a chronic course (≥ 12 months). A subset of EV patients (*n* = 20) had undergone comprehensive HPV genotyping in a previous study [17], whereas the remaining EV cases fulfilled established diagnostic criteria without molecular characterization. For each participant, samples were categorized as flat wart or as clinically normal skin from unaffected areas; normal skin was obtained from anatomically matched sites to the wart sample within the same individual.

The analysis included 135 samples: 42 from EV patients (17 flat warts, 25 normal skin) and 93 from NEV individuals (21 flat warts, 72 normal skin). Demographic and anatomical data were extracted from medical records and grouped as head/neck, trunk, and limbs (Table 1).

**Table 1:** Demographic characteristics and topography of the lesions.

Group	*n*	Age (years)	Male (%)	Female (%)	Head and neck (%)	Trunk (%)	Limbs (%)
Normal skin—EV	25	49.4 ± 14.2	84.0%	16.0%	52.0%	40.0%	8.0%
Flat wart—EV	17	32.5 ± 14.5	41.2%	58.8%	11.8%	47.1%	35.3%
Normal skin—NEV	72	75.7 ± 10.5	55.6%	44.4%	61.1%	23.6%	15.3%
Flat wart—NEV	21	43.6 ± 23.9	57.1%	42.9%	28.6%	19.0%	52.4%

*Note:* Demographic characteristics and anatomical distribution across diagnostic groups. Age is presented as mean ± SD. Sex distribution is shown as percentage of males (M) and females (F). Anatomical distribution is expressed as percentage of lesions in head/neck, trunk, and limbs.

Abbreviations: EV, epidermodysplasia verruciformis; NEV, non‐epidermodysplasia verruciformis.

### 2.2 Immunohistochemistry

Tissue Sections (3–4 µm) from selected FFPE blocks were mounted on silanized slides, dried overnight at 37°C, deparaffinized in xylene, and rehydrated in graded ethanol. Antigen retrieval used citrate buffer (pH 6.0) in a pressure cooker (95°C, 20 min). Endogenous peroxidase activity was blocked with 3% hydrogen peroxide in methanol (10 min).

Slides were incubated overnight at 4°C with primary antibodies: anti‐TLR3 (1:100, Abcam), anti‐TLR4 (1:150, Santa Cruz Biotechnology), anti‐TLR5 (1:100, Novus Biologicals), and anti‐TLR9 (1:200, Santa Cruz Biotechnology). Detection employed a polymer‐based system (REVEAL Biotin‐Free DAB Detection System) with 3,3′‐diaminobenzidine as chromogen. Slides were counterstained with Harris hematoxylin, dehydrated, cleared, and mounted with synthetic resin.

Positive controls included normal skin (TLR3, TLR4), molluscum contagiosum (TLR3, TLR5), and cutaneous leishmaniasis (TLR9) [10, 18, 19]. Negative controls substituted the primary antibody with isotype‐matched nonimmune serum. The observed cellular distribution and compartment localization (predominantly epidermal keratinocytes) were concordant with published patterns for these targets [20], supporting specificity in our material. Analysis was restricted to the epidermal keratinocyte compartment; the dermal inflammatory infiltrate was not phenotyped and was typically sparse.

### 2.3 Digital Image Acquisition and Quantification

Slides were digitized at 40× magnification using the Aperio ScanScope. TLR expression was quantified in ImageJ by color deconvolution and automatic thresholding. Five epidermal fields per sample were analyzed, avoiding artifacts. Results were expressed as percent stained area. Quantification was performed by a blinded researcher and reviewed by a dermatopathologist.

### 2.4 Statistical Analysis

Analyses used Python and StatsModels. Mann–Whitney U tests followed Shapiro–Wilk assessment. Kruskal–Wallis tests assessed associations with sex and site. Multivariate linear regressions adjusted for diagnosis, age, sex, and site. Significance was set at *p* < 0.05.

## 3 Results

A total of 135 samples were analyzed across four groups: normal skin – EV (n = 25), flat wart – EV (n = 17), normal skin – NEV (n = 72), and flat wart – NEV (n = 21). EV patients were younger, with flat wart samples averaging 32.5 ± 14.5 years and normal skin 49.4 ± 14.2 years, while NEV individuals were older overall (75.7 ± 10.5 years in the normal skin group).

**Figure 1:**
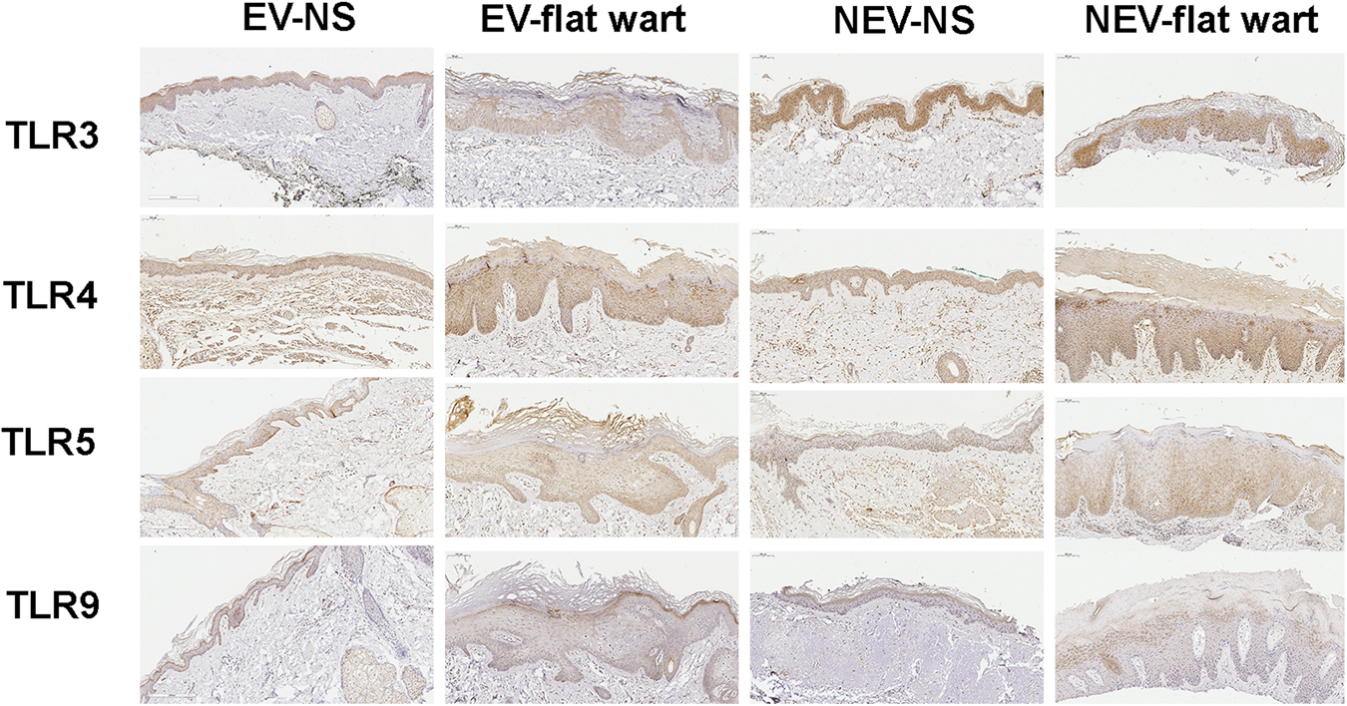
Low‐power overview immunohistochemistry for Toll‐like receptors in skin. Panels show TLR3, TLR4, TLR5, and TLR9 in normal skin (NS) and flat warts from individuals with epidermodysplasia verruciformis (EV) and without EV (NEV). Representative epidermal fields are displayed; staining was performed on FFPE sections with 3,3′‐Diaminobenzidine (DAB) chromogen and Harris hematoxylin under uniform conditions. Immunoreactivity localizes predominantly to keratinocytes, including koilocytotic areas in warts. EV, epidermodysplasia verruciformis; NEV, non‐EV; NS, normal skin.

**Figure 2:**
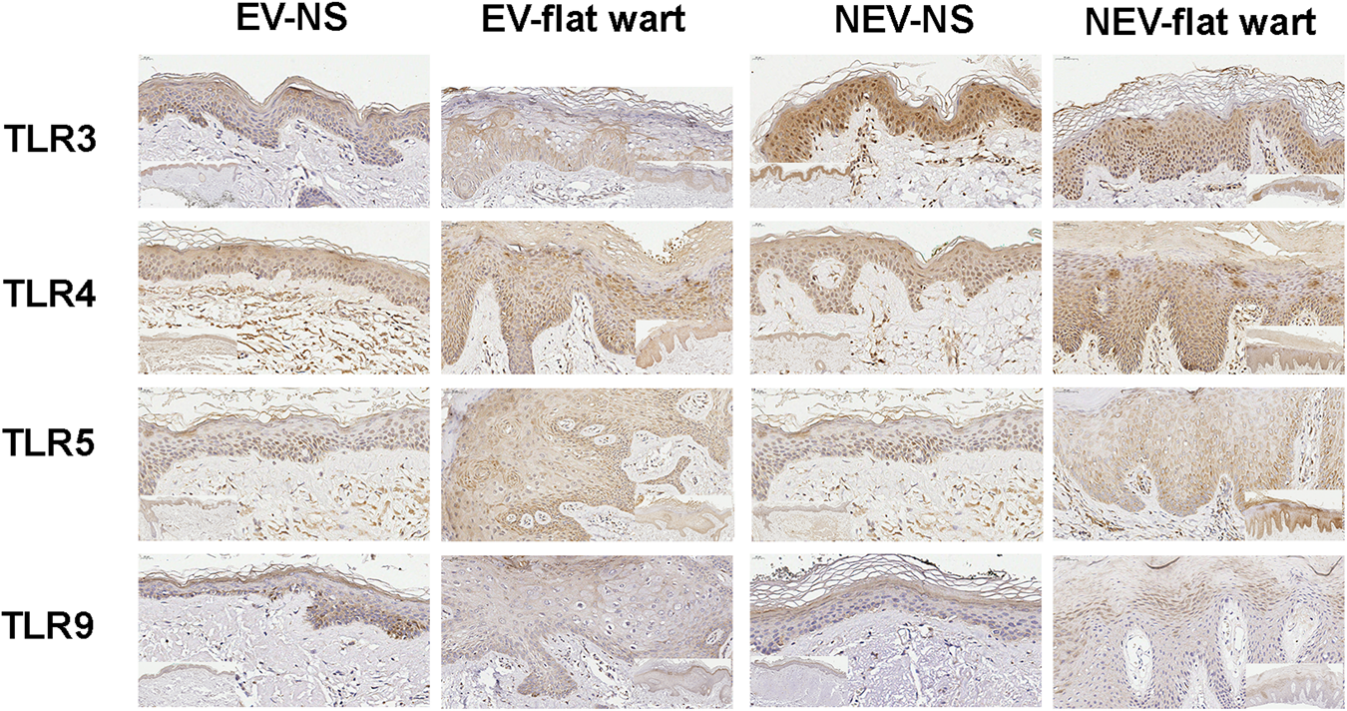
Higher‐magnification views of the epithelial compartment in flat warts. Panels depict keratinocyte staining for TLR3, TLR4, TLR5, and TLR9. Images were processed and acquired under identical conditions; analysis focused on the epidermal keratinocyte compartment, and the dermal inflammatory infiltrate—typically sparse—was not phenotyped. Scale bars (top–left). Insets show low‐power overviews of matched normal skin and flat warts.

TLR3, TLR4, TLR5, and TLR9 expression was detected in the epidermis of both normal skin and flat warts from EV and NEV individuals. In EV flat warts, immunostaining was reduced in koilocytotic keratinocytes compared with adjacent non‐koilocytotic keratinocytes—most notably for TLR3—whereas NEV flat warts exhibited stronger staining in koilocytotic areas (Figure 1). High‐magnification views (Figure 2) show that staining predominated in epidermal keratinocytes; dermal inflammatory cells were scarce and were not phenotyped.

**Figure 3:**
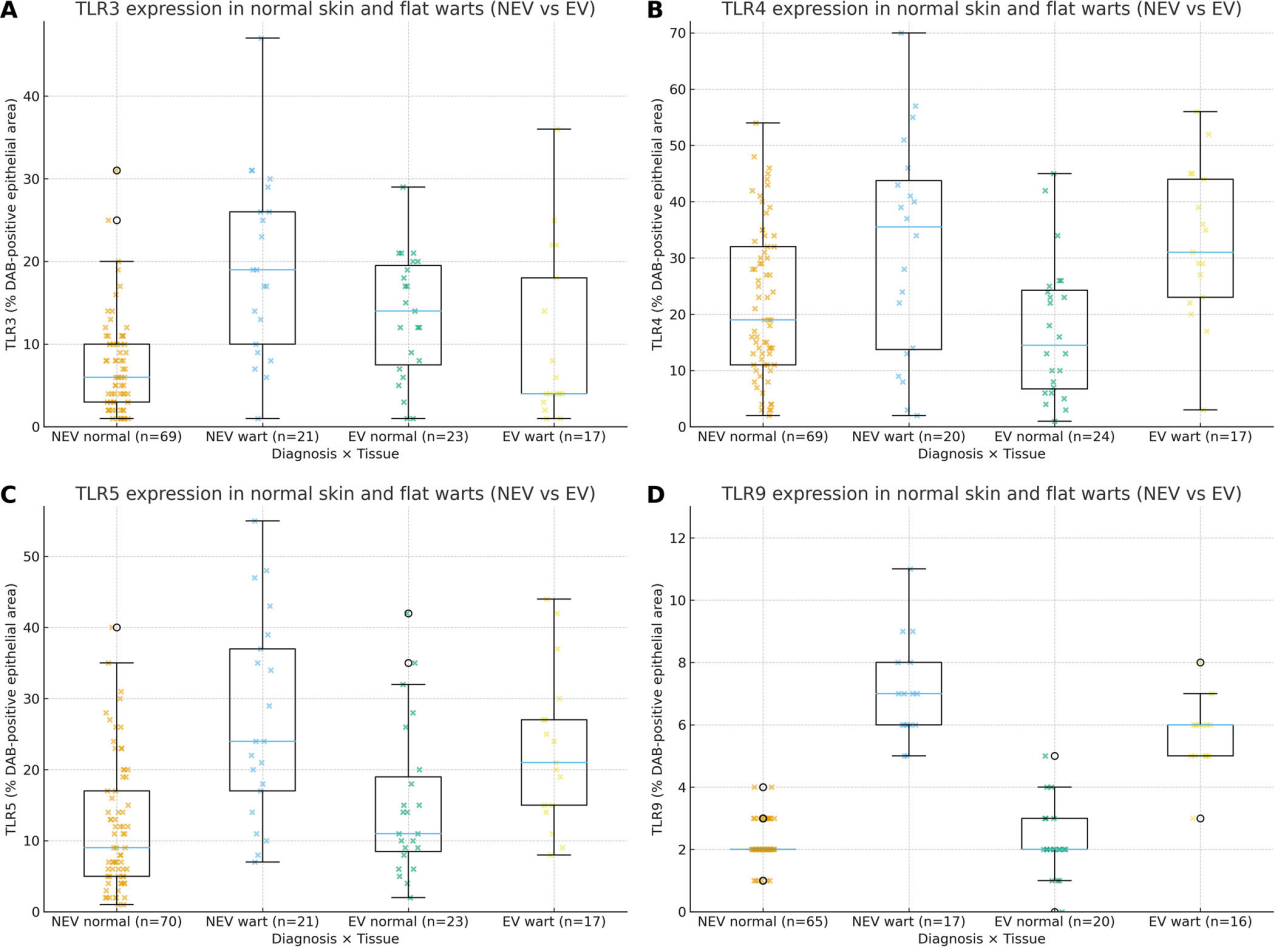
TLR expression comparison in normal skin and flat warts from NEV and EV. Boxplots display the percentage of DAB‐positive epithelial area for four subgroups on the same y‐axis: NEV normal, NEV wart, EV normal, and EV wart (sample sizes shown on the x‐axis). In NEV, TLR3 is higher in warts than in normal skin, whereas no wart‐vs.‐normal difference is observed in EV; across diagnoses, EV shows lower TLR3 and TLR9 than NEV. Pairwise comparisons used the Mann–Whitney U test; exact *p* values are reported in the Results. Panels: (A) TLR3, (B) TLR4, (C) TLR5, (D) TLR9.

Kruskal–Wallis testing indicated significant variation among groups for TLR3 (*p* < 0.001) and TLR9 (*p* < 0.001), but not for TLR4 (*p* = 0.485) or TLR5 (*p* = 0.071). Mann–Whitney U tests revealed lower TLR3 and TLR9 expression in EV patients compared to NEV controls (Figure 3). For TLR3, median values were 4.0% in EV versus 19.0% in NEV (*p* = 0.008), with similar differences in normal skin (*p* < 0.001). TLR9 showed median values of 5.6% in EV versus 7.0% in NEV (*p* = 0.007), extending to normal skin (*p* = 0.008). TLR4 and TLR5 showed no significant group differences (*p* = 0.927 and *p* = 0.499, respectively).

Within‐group comparisons showed that NEV patients had significantly higher TLR3 expression in flat warts than in normal skin (*p* = 0.020), suggesting an adaptive response to viral infection. This upregulation was absent in EV patients (*p* = 0.167). For TLR9, no significant differences were observed between flat warts and normal skin in either group.

No sex‐based differences in TLR expression were found. Expression heterogeneity was notable, with the highest coefficient of variation for TLR3 in the EV group (99.6%), suggesting variable receptor regulation across samples.

Multivariate models including diagnostic group, age, sex, and anatomical site as covariates were constructed for each TLR. EV diagnosis remained significantly associated with lower TLR3 expression (*p* = 0.025), while no other variables reached significance. For TLR9, NEV status (*p* = 0.031) and age (*p* = 0.011) were significant predictors, with the model showing substantial explanatory power (adjusted *R*² = 0.493). TLR4 and TLR5 models showed limited significance and low explanatory power. Sex had no significant effect in any model.

## 4 Discussion

This study demonstrates significantly reduced TLR3 and TLR9 expression in both normal skin and flat warts of Epidermodysplasia Verruciformis (EV) patients compared to immunocompetent individuals (NEV). Because we assessed expression rather than signaling or virological outcomes, implications for β‐HPV control or carcinogenesis cannot be inferred from these data.

In contrast, NEV individuals exhibited higher TLR3 in flat warts than in matched normal skin, supporting a wart‐associated induction. EV patients did not show this induction and maintained low TLR3 across both tissue types, consistent with the absence of wart‐associated TLR3 upregulation observed in NEV.

TLR4 and TLR5 remained stable between groups. This aligns with reports of TLR expression in human keratinocytes and with their detection in cutaneous viral lesions [4, 10] and, within this data set, indicates no between‐group differences for these receptors.

TLR9 showed the lowest detectability overall, with a more pronounced reduction in EV patients. Notably, even clinically normal skin from EV patients exhibited lower TLR3/TLR9 than NEV controls, indicating a baseline difference rather than a purely lesion‐associated change.

Together, TLR3 and TLR9 delineate an EV‐specific epithelial expression profile. These reduced expression levels suggest differences in epithelial innate‐sensing capacity; however, whether these differences are functionally relevant to β‐HPV sensing or contribute to viral persistence and carcinogenesis was not tested here and requires further investigation. EV also exhibits abnormalities in epidermal differentiation markers (e.g., cytokeratins, involucrin, filaggrin, E‐cadherin) compared with non‐EV warts [12], suggesting disease‐specific epithelial programs; possible links between differentiation state and TLR abundance were not assessed here.

Mechanistically, TLR3 recognizes double‐stranded RNA in model systems; dsRNA production by cutaneous HPV has not been established. We quantified epithelial TLR protein expression; functional activation was not assessed. Epithelial TLR pathways can be biologically relevant in DNA‐virus settings [21]. TLR3 expression has been reported in verruca vulgaris and molluscum contagiosum [10] and associated with HPV‐related cervical disease [14]. In our cohort, NEV warts showed higher TLR3 than matched normal skin, whereas EV lacked this wart‐associated induction—an epithelial expression phenotype rather than evidence of anti‐HPV activity. In keratinocytes, engagement of TLR pathways can elicit type I interferon and NF‐κB programs [1, 4]. Multiple explanations remain plausible—including epigenetic regulation or host background—and warrant testing in keratinocyte systems.

For TLR9, keratinocytes respond to CpG stimulation by inducing inflammatory mediators, indicating functional signaling capacity [4]. In cutaneous HPV lesions, context‐dependent patterns have been described: increased TLR9 and IRAK1 with unchanged TLR7/IRF7 (interferon regulatory factor 7) in common warts, and higher TLR3/TLR9 in warts than in normal skin [10, 11]. Our findings extend this cutaneous context by demonstrating marked TLR9 reductions in EV patients across both normal skin and flat warts, representing a distinct epithelial expression phenotype. Beyond skin, TLR9 promoter polymorphisms have been associated with risk and prognosis in gastric cancer cohorts [22], although these findings do not address epithelial protein expression.

In contrast to the altered expression of endosomal TLRs (TLR3 and TLR9), TLR4 and TLR5 are surface‐expressed receptors [1, 2]. Correspondingly, epidermal TLR4 and TLR5 levels were stable between EV and NEV within our data set, in line with reports of constitutive expression in human keratinocytes and detection in cutaneous viral lesions [4, 10].

While our findings are specific to cutaneous HPV disease, studies in other epithelial tumors have reported associations between tumor‐cell TLR3 and clinical outcomes—favorable prognosis in triple‐negative breast cancer and apoptosis upon experimental activation in non‐small‐cell lung cancer—highlighting clinical interest in epithelial TLR patterns while underscoring differences in disease context [23, 24]. For TLR9, studies in common warts indicate context‐dependent behavior [10, 11]; our data address epidermal expression only.

Several limitations should be acknowledged. Despite careful case selection, our modest sample size may limit generalizability. While immunohistochemical detection assesses protein abundance at the tissue level, it does not provide direct evidence of functional signaling activity; observed TLR expression should not be interpreted as evidence of responsiveness to viral ligands. This caveat particularly applies to TLR3 in NEV warts, where we did not evaluate downstream readouts (e.g., IRF3 phosphorylation, interferon‐stimulated genes). Thus, higher TLR3 expression should not be construed as evidence of pathway activation.

The absence of molecular profiling constitutes another limitation. Host factors, including genetic variants, regulatory microRNAs, and epigenetic modifications, were not evaluated, constraining conclusions about the mechanistic basis of reduced TLR expression in EV. Although TLR polymorphisms have been linked to HPV‐associated disease [25, 26], their specific role in EV‐related TLR dysregulation remains undefined.

Future investigations should incorporate functional assays to test whether differential receptor expression corresponds to downstream signaling competence. Ex vivo stimulation with TLR agonists could probe signaling capacity in EV versus NEV keratinocytes. Clinical implications, if any, would require dedicated studies, ideally integrating HPV genotyping and host genetic factors (e.g., TMC6/TMC8/CIB1). This combination—lower TLR3/TLR9 and absence of wart‐associated induction in EV—defines a precise, testable framework for subsequent functional work on pathway competence in keratinocytes.

## Author Contributions

**Luis Alberto Ribeiro Fróes Jr.:** data curation, methodology, formal analysis, investigation, validation, visualization, writing – original draft. **Cibele Conceição dos Apóstolos Pereira:** conceptualization, data curation, investigation, and methodology. **Lana Luiza da Cruz Silva:** data curation and investigation. **Naiura Vieira Pereira:** data curation and investigation. **Walmar Roncalli Pereira de Oliveira:** conceptualization, methodology, data curation, investigation, and supervision. **Mirian Nacagami Sotto:** conceptualization, data curation, formal analysis, funding acquisition, investigation, methodology, project administration, resources, supervision, validation, and writing – review and editing.

## Conflicts of Interest

The authors declare no conflicts of interest.

## Data Availability

The data that support the findings of this study are available on request from the corresponding author. The data are not publicly available due to privacy or ethical restrictions.
